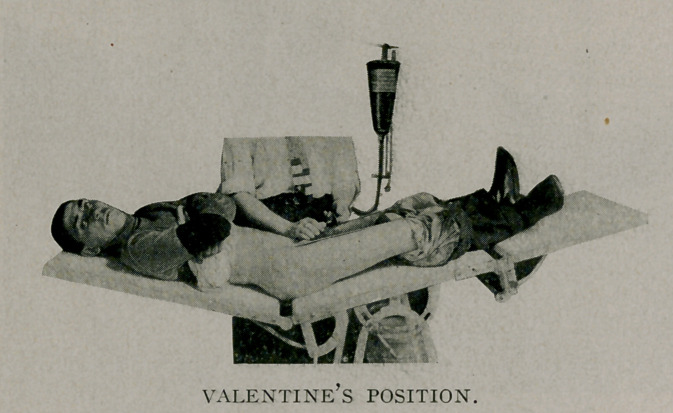# The Treatment of Subacute Gonorrhoea and Complications

**Published:** 1911-11

**Authors:** Charles W. Bethune

**Affiliations:** 131 Allen Street, Buffalo, N.Y.


### The Treatment of Subacute Gonorrhoea and Complications

CONTINUED FROM OCTOBER.

By CHARLES W. BETHUNE, M.D.
131 Allen Street, Buffalo, N. Y.

FOLLOWING the termination of the acute posterior symptoms, the so-called subacute stage ensues. It is characterized by a marked increase in the discharge over that of the acute posterior stage, but urination is only slightly painful and the nocturnal erections are not so distressing. Except in very mild cases, turbidity of the second glass of urine persists. In mild cases the purulent excretion of the posterior urethra may be so scanty that the bladder urine is not contaminated and the first jets of urine wash out all pus, the second glass in this case being clear. This fact cannot be repeated too often for it is a common source of error. Turbidity due to phosphates must not be forgotten and is to be eliminated by the addition of a few drops of nitric acid to each glass of urine.

Irrigations are to be continued but as yet no attempt must be made to fill the bladder. The anterior urethra may be gently distended with the irrigating fluid, the patient's sensations being the best guide as to its advisability.

The posterior infection tends towards spontaneous recovery and in the vast majority of cases, unless the prostate and vessicles are involved it terminates in two or three weeks. If cloudiness of the second glass persists more than two or three weeks and distention of the urethra does not cause distress, an attempt to force the cut-off muscle may then be made. After the anterior urethra has been flushed out several times, the tip is gently pressed into the meatus and the patient told to try to urinate, (relax the sphincter). This manoeuver is generally rewarded by the rapid filling of the bladder. If this method fails, the patient is requested to sit on a hard chair, the tuber ischii resting on the edge. In this position one but rarely fails to fill the bladder. When both these methods fail, a second or third attempt is almost invariably successful. When the bladder is filled, the posterior urethra is twice irrigated, by the inflow and the outflow. This method is usually followed by a speedy clearing of the second glass.

Filling the bladder through a catheter during the subacute stage or, for that matter, the introduction of any urethral instru-

BETHUNE TIP.

ment is to be emphatically condemned because of the danger of lighting up an epididymitis. The danger of epididymitis is at its height during the first two weeks of the subacute stage, after that it steadily diminishes. Epididymitis will occur in some cases in spite of all precautions, but it is inadvisable to invite its occurence by over enthusiastic treatment.

Prostatitis and vesiculitis when they complicate a case, usually ensue within a day or two after the onset of the posterior symptoms. The symptoms of these complications are those of posterior invasion, only more accentuated. In mild cases they may cause nò accentuation of the posterior symptoms, but the severer forms, those enumerated in the previous paper together with painful bloody nocturnal seminal emissions. The condition is diagnosed by rectal palpation, the prostate and vessicles being swollen, hot and tender.

In the acute stage the treatment is the same as that of the posterior involvement. In the subacute stage massage must be religiously avoided, the danger of lighting up an epididymitis being great. Vaccines are the most potent weapon in the therapeutic arsenal against this complication.

An initial dose of 5,000,000 stock culture should be injected into the subcutaneous tissues of the thigh as soon as the acute

METHOD OF HOLDING TIP.

symptoms subside. The injection is to be repeated every three days, doubling the dose each time until a reaction occurs or the patient recovers. The reaction is characterized by a temperature of 101-105 Fahrenheit together with malaise on the day following the injection. In about half the cases no reaction occurs, but in spite of its absence the condition rapidly improves. On the day after the reaction there is usually a marked improvement in the symptoms, which is permanent. Massage of the prostate and vesicles should be employed only during chronic gonorrhoea and will be discussed later.

Abscess of the prostate is a rare complication of prostatitis. Although it usually ruptures into the urethra and causes no further trouble, the danger of rupture in other directions with resultant fistulæ and sinuses indicate immediate perineal incision and drainage. The same treatment applies to the still rarer abscesses of Cowper's gland.

It is impossible to differentiate a severe posterior invasion from prostatitis clinically, and so far as treatment is concerned it is unnecessary.

Epididymitis is the most common of the severe complications of gonorrhoea, severe in that it causes the patient considerable suffering and confines him to bed for a couple of weeks, not to mention the possibility of subsequent sterility. Infection of the epididymis is preceeded by inflammation of the vas deferens. The symptoms of vasitis are slight, at the most tenderness over the point where the cord passes through the external ring. When a patient complains of tenderness at this point he should be ordered to bed at once and the scrotum supported by a broad strip of adhesive stretched over the anterior surfaces of the thighs. If this is done many an attack of epididymitis will be avoided.

VALENTINE'S POSITION.

Epididymitis ensues a day or two after the onset of vasitis. The patient first notices that the testicle feels heavy and that there is a tender indurated nodule most often in the lower, more rarely in the upper pole of the epididymis. The epididymis then begins to swell until the testicle is surrounded by a horseshoe-shaped body several inches in thickness. If the patient attempts to stand the pain is excruciating and there is no difficulty in persuading him to remain in bed. The scrotum is to be supported upon the adhesive plaster bridge, mentioned above. Poultices applied to the scrotum give marked relief, ice applications are to be avoided as there are a number of cases reported in which gangrene followed their use. Rubifacient antiseptic ointments are frequently smeared on cloths and applied to the scrotum. I have found the following formula to be very soothing.

| | | |
|---|---|---|
| R | Menthol | gr. XV |
| | Ung. Belladonnæ | gr. XX |
| | Ung. Crede | gr. XXX |
| | Ichthyol | gr. LX |
| | Vaseline, Q. S. Ad. | oz. I |

The swelling subsides in about ten days, often leaving an indurated nodule about the size of a pea at the site of the original nodule mentioned above. Although this nodule does no harm, unless it is so located that it obstructs the orifice into the vas, it is often a source of great anxiety to the patient. It may gradually disappear, but in case it persists and the patient insists on its removal, the scrotum may be opened and a cris-cross incision of the tunica albuginia made over it. This manoeuver is almost invariably successful.

If the swelling of the epididymis does not quickly resolve, the testicle should be strapped. Envelop the affected half of the scrotum in a square of gauze, encircle the upper pole of the testicle with the thumb and index fingers, pressing the testicle into the bottom of the scrotum. Bind a strip of adhesive above the testicle so tightly that it cannot slip through. Starting at this so-called choker pass strips of adhesive around under the testicle to the opposite side of the choker until the entire testicle is covered, then apply a second choker over the ends of the strips. When strapped, the size of the testicle rapidly diminishes, necessitating the renewal of the strapping every second day. The strapped testicle should be supported by a suspensory.

Inguinal adenitis, or bubo, is a common complication of acute and subacute gonorrhoea. Painting with tincture of iodine is as good as any treatment. Suppuration occurs but rarely and of course requires incision and drainage.

Balanitis is avoided by scrupulous cleanliness of the praeputial cavity. Its treatment consists of frequent cleansing with peroxide followed by the application of a dusting powder. Inflammatory phimosis indicates thorough flushing of the praeputial cavity with the permanganate solution together with prolonged soaking in very hot water. If this does not reduce the swelling in a week dorsal incision is indicated. Periurethral abscess indicates early incision, care being taken not to wound the urethra.

Stricture due to a previous attack of gonorrhoea does not seriously interfere with irrigation treatment provided that its calibre is above 15 French. A calibre below 15 French is a marked obstacle, especially if it is situated in the penile urethra. Dilatation during the acute and subacute stages is both painful and dangerous, there is also a constant danger of occlusion of the already narrowed lumen from swelling of the mucosa. If acute retention occurs, injections of adrenalin chloride 1-100,000, and hot sitz baths and leaching of the perineum should be tried before catheterization. These failing, catheterization is our only resort, under anaesthesia if necessary. I have occasionally been obliged to employ gradual dilitation during the subacute stage and as yet have had no more serious results than epididymitis, but I always do it in fear and trembling.

In uncomplicated cases the discharge of the subacute stage steadily diminishes under the irrigation treatment until only a morning drop is present. This morning drop is the characteristic of the chronic stage which when improperly treated or not treated at all may last for years. The treatment of chronic gonorrhoea will be discussed in a subsequent paper.

## Figures and Tables

**Figure f1:**
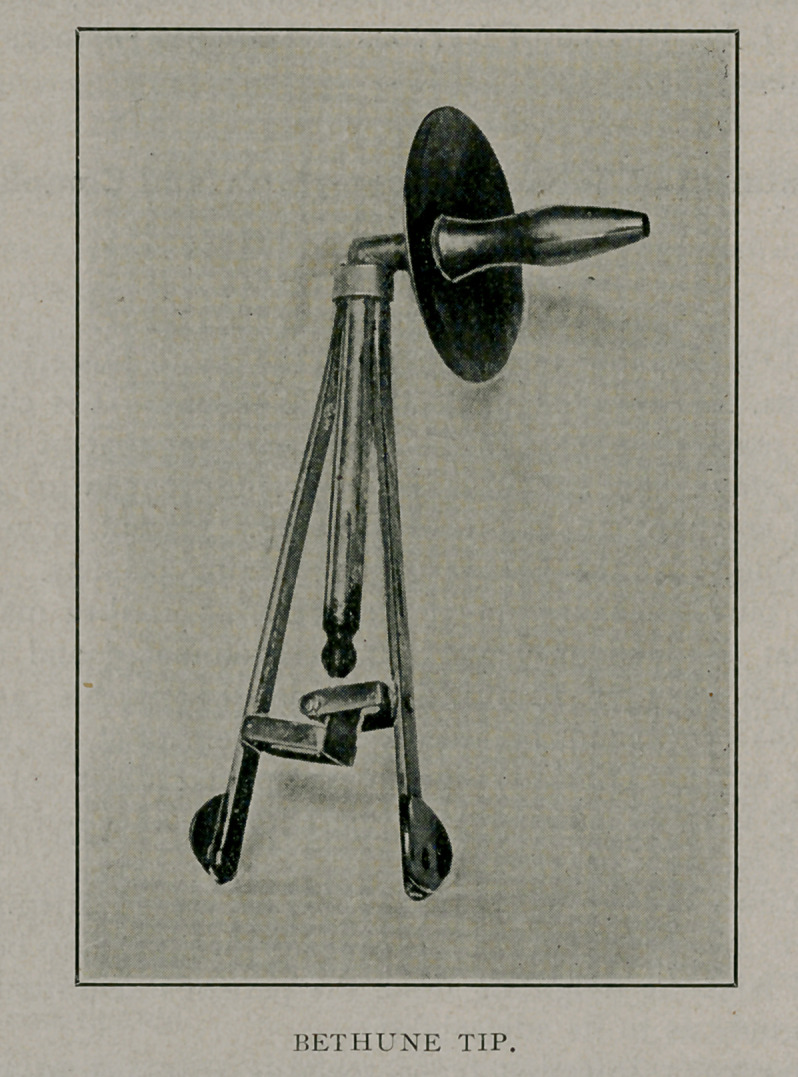


**Figure f2:**
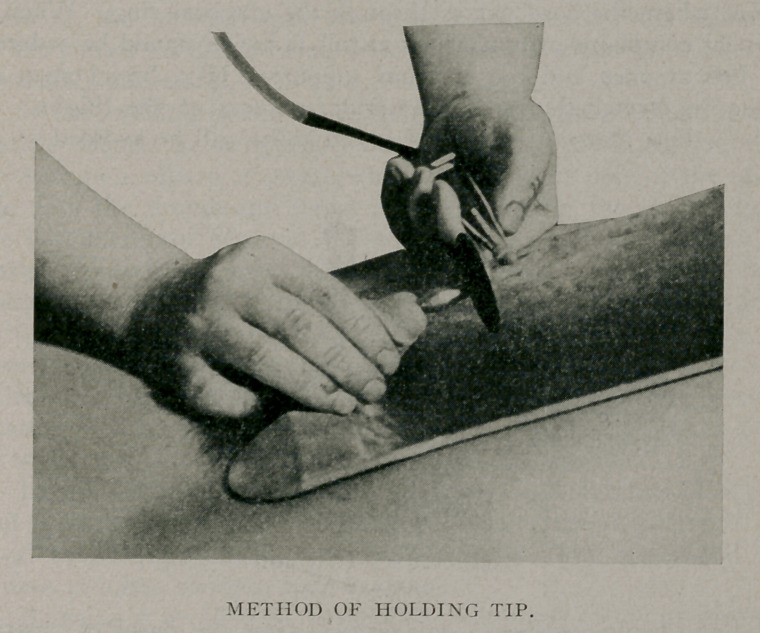


**Figure f3:**